# Prevalence of Aflatoxin Contamination in Herbs and Spices in Different Regions of Iran

**Published:** 2017-11

**Authors:** Payam KHAZAELI, Mitra MEHRABANI, Mahmoud Reza HEIDARI, Gholamreza ASADIKARAM, Moslem LARI NAJAFI

**Affiliations:** 1. Pharmaceutics Research Center, Institute of Neuropharmacology, Kerman University of Medical Sciences, Kerman, Iran; 2. Dept. of Pharmaceutics, Faculty of Pharmacy, Kerman University of Medical Sciences, Kerman, Iran; 3. Herbal and Traditional Medicines Research Center, Kerman University of Medical Sciences, Kerman, Iran; 4. Endocrinology and Metabolism Research Center, Institute of Basic and Clinical Physiology Sciences, Kerman University of Medical Sciences, Kerman, Iran

**Keywords:** Mycotoxins, HPLC, Survey

## Abstract

**Background:**

Mycotoxins are natural toxins, produced by several fungal species and are associated with morbidity or even mortality in animals, plants, and humans. In this study, 120 samples of herbs and spices in both bulk and packaged forms were prepared in order to measure aflatoxin level in different regions of Iran

**Methods:**

The aflatoxin was extracted during Mar to May 2015, using 80% methanol and then purified via immunoaffinity column. Measurements were performed, using high-performance liquid chromatography, equipped with a fluorescence detection system at excitation and emission wavelengths of 365 and 435 nm, respectively.

**Results:**

The highest prevalence of aflatoxin contamination in food products was attributed to aflatoxin B1 (30.8%). In addition, the highest prevalence of aflatoxin contamination was reported in red pepper (100%). Examination of effective factors indicated the substantial impact of moisture on aflatoxin level (*P*=0.046).

**Conclusion:**

Even at low levels of aflatoxin, contamination could be a serious threat, given the prevalent use of spices (either raw or not) as ingredients in food preparation. Therefore, regular monitoring of spices, especially chili pepper, is highly recommended.

## Introduction

Mycotoxins are natural toxins, produced by several fungal species and are associated with morbidity or even mortality in animals, plants, and humans. These compounds have diverse chemical structures and a low molecular weight. Mycotoxins are often found in a large number of agricultural and food products throughout the world.

In different stages such as the production, harvest, transport, and storage of agricultural products, mycotoxins can result in the contamination of human food or animal feed. Nevertheless, these fungi are not endemic to specific geographical areas or climates. In fact, fungal growth and toxin production occur only if the environment and conditions are suitable (1).

Mycotoxins constitute an important group of toxic compounds, produced by certain types of fungi (2). Aflatoxins are the most important group of mycotoxins, produced by different *Aspergillus* species, i.e., *A. flavus*, *A. parasiticus*, and *A. nomius* (3). Among recognized aflatoxins, Aflatoxin B1 is considered to be the most toxic (4). In fact, aflatoxin was included in the list of cancer-causing agents by the International Agency for Research on Cancer in 1987 (5).

Improper storage, drought, and high humidity can lead to an increase in the growth of *Aspergillus* species in herbs and spices (6). Spices are mostly produced in humid and tropical countries, where a suitable environment for fungal growth is provided (7). The European Union has established maximum tolerable limits in spices at 10 mg/kg for total aflatoxins and 5 mg/kg for aflatoxin B1 (8).

The aim of this study was to investigate the prevalence of aflatoxin contamination in herbs and spices and determine the effective factors in different regions of Iran. Continuous assessment and control of fungal contamination in medicinal herbs and spices, used by families and food industries, can guarantee the health of consumers and promote competition in the international market.

## Materials and Methods

### Sampling

The study protocol was reviewed and approved by the Pharmaceutical Research Center, Faculty of Pharmacy, Kerman University of Medical Sciences (No: 93/1501).

Overall, 120 samples of herbs and spices in both bulk and packaged forms (104 and 16 samples, respectively) obtained randomly from retail sales points in different regions of Iran, i.e., Tabriz, Kerman, Tehran, Bandar Abbas, and Sari were analyzed during Mar to May 2015. 100 g of each sample have been immediately brought to the laboratory at 4–6 °C and analyzed.

### Sample preparation

The samples were prepared, using a laboratory grinding mill and 40-mesh sieve passed. In order to achieve a uniform composition, the samples were mixed by a mixer.

### Standards and reagents

All chemicals with the exception of methanol and acetonitrile were laboratory-grade compounds and purchased from Merck, Germany. Standards including AFG2, AFG1, AFB2, and AFB1 were purchased from Sigma (Germany).

### Apparatus

Liquid chromatography (LC) was performed, using a reversed-phase high-performance LC (HPLC) system (Waters 2695, USA), equipped with a Gilson Workstation (Gilson GX-271 Aspec, USA) and a fluorescence detector (Waters 474, USA). The capital HPLC column was C18 (15 cm×4.6 mm, 5 μm). Moreover, aflatoxin immunoaffinity columns (IACs) were purchased from R-Biopharm (Darmstadt, Germany).

### Extraction method

For this purpose, 10 gr of the ground sample was mixed in 60 ml of methanol 80% for 30 min, using a shaker and then filtered; the product was then centrifuged for 30 min and re-filtered. Finally, 0.25 ml Tween was added to 5 ml of the filtered extract and stirred for 2 min.

### Aflatoxin separation using IAC

For this purpose, 3.1 ml of the filtered extract was diluted in 9.9 ml of distilled water and filtered, using a microfiber filter. Then, 12.6 ml of the extract was used for IAC, preconditioned with 10 ml of phosphate-buffered saline (rate of 14 drops per min). After passing the extract through the column, the column was rinsed twice with 15 ml of water and then dried. Aflatoxin was collected in the vial, using 1.25 ml of methanol and diluted with 1.75 ml of deionized water. Finally, 100 μl of the solution was injected into the HPLC system.

### Aflatoxin measurement by HPLC

Aflatoxin level was determined, using HPLC, equipped with a fluorescence detection system, using C18 silica gel column and Kobra cell derivatization at excitation and emission wavelengths of 365 and 435 nm, respectively. The mobile phase consisted of water-acetonitrilemethanol (60:20:30 v/v/v) containing 120 mg/L KBr and 350 μl HNO3 4M, with a flow rate of 1 mL per min and an injection volume of 100 μL.

### Calibration curve

A calibration curve was prepared by using the working standard solutions. The tertiary stock standard was used to prepare the working standard solutions by pipetting appropriate volumes of a mixed standard solution (1000 ppm for B1and G1 and 200 ppm for B2 and G2) into a 4ml vial and take to 2 ml volume in the way that the final concentration of solution be 100 ppb for B1 and G1 and 20 ppb for B2 and G2, respectively. The working standard solution was prepared by pipetting appropriate volumes of prepared stock solution to 4ml vial and take to 2mlvolume in the way that final concentration of solution is 7.2, 5, 3.6, 2.8, 2, 1.2, 0.4 ppb for B1 and G1, respectively. A seven-point calibration curve was built for each type of aflatoxin. The calibration curve was constructed for the analysis to check the pilot for linearity (R2= 0.999) and was used for quantification of aflatoxin. If the content of toxins in the sample was outside the calibration range, a more appropriate calibration curve was prepared, or the injection solution for LC analysis was diluted to an aflatoxin concentration appropriate for the established calibration curve.

### Quality assurance

In addition to using validated methods, internal and external quality control experiments were performed. Regarding internal quality control, the accuracy and precision of the methods were verified. For this purpose, AFB1, B2, G1, and G2 recoveries were recorded by analyzing a blank sample, spiked at 4 ng/g for AFB1 and AFG1 and 1 ng/g for AFB2 and AFG2, recovery rate for AFB1 was 50%–70% and the average coefficient of variation was 5.4%. The aflatoxin level was corrected, according to the recovery value. LOD and LOQ for AFB1 were 0.033 ppb and 0.1ppb, respectively.

### Statistical analysis

For statistical analysis, data were entered to SPSS (Chicago, IL, USA) Ver. 18. The qualitative data were analyzed and the correlation between nominal variables was evaluated, using Chi-square. After confirming the normality of quantitative data, ANOVA test was applied for making comparisons. In case the data were not normally distributed, non-parametric tests were applied. *P-*value less than 0.05 was considered statistically significant.

## Results

Among 120 tested samples, 37 (30.8%) were contaminated with aflatoxins (range: 0.2–57.5 μg/kg); based on the findings, all these samples were contaminated by AFB1 (0.7–57.5 μg/kg) (Table 1). Overall, in 16 (13.3%) and 12 (10%) samples, AFB1 and total aflatoxin levels surpassed the standard limits, proposed by the European Union (5 and 10 μg/kg, respectively).

**Table 1:** Aflatoxin B1 content of herb and spice samples

	***n***	***Positive samples N (%)***	***Number of samples surpassing the European Union limit (%)***	***Range of AFB1 concentration in the positive samples (μg/kg)***
Sumac	9	0(0)	0(0)	ND^*^
Chamomile	7	100(7)	57(4)	2.0–16.6
Green cumin	10	0(0)	0(0)	ND
Black cumin	6	0(0)	0(0)	ND
Turmeric	10	80(8)	0(0)	1.3–4.2
Bay leaf	7	0(0)	0(0)	ND
Black pepper	7	71(5)	14(1)	0.7–8.4
Red pepper	10	100(10)	100(10)	15.5–57.5
Sage	2	50(1)	0(0)	2.5–2.5
Basil seeds	6	0(0)	0(0)	ND
Thyme	10	10(1)	0(0)	2.2–2.2
Mint	9	0(0)	0(0)	ND
Ginger	9	55(5)	11(1)	1.9–39.8
Fennel	9	0(0)	0(0)	ND
Cinnamon	9	0(0)	0(0)	ND
Total	120	37	16	0.7–57.5

* ND: Not detected

Additionally, B2 aflatoxin contamination (0.2–3.4 μg/kg) was detected in 17 (14.2%) samples. In addition, aflatoxin G1 contamination (1.8–31.2 μg/kg) was reported in 4 (3.3%) samples, and aflatoxin G2 contamination (range: 2.1–2.1 μg/kg) was detected in 1 (0.83%) sample. A significant toxic level in herbs and spices and AFB1 was the most common agent of contamination among different types of aflatoxins.

Examination of factors affecting aflatoxin level showed a significant difference between the samples in terms of moisture (*P*=0.046). In addition, aflatoxin level was not significantly influenced by the packaging of the samples (*P*=0.578).

Growth and harvest of spices in tropical and sub-tropical countries are not standard. In fact, high level of contamination in spices, especially red pepper, might be rooted in improper growth and harvest conditions. In the present study, contamination of the samples was at its initial stage, which would definitely increase by time. Therefore, the lower level of infection at early stages would be indicative of the higher safety of the final product.

## Discussion

Considering the significance of herbs and spices due to their medicinal and nutritional benefits, a great deal of attention has been paid to the measurement of aflatoxin, as a threatening agent which can appear in different stages of cultivation, harvest, processing, and storage of agricultural products.

Similarly, aflatoxin level was strongly influenced by the moisture content of raw compounds (9).

The amount of aflatoxin in 12 different powdered spices (i.e., 5 cardamoms, 5 red pepper, 8 pepper, 5 cloves, 7 cumin, 5 turmerics, 5 ginger, 5 mustard, 10 nutmeg, 12 paprika, 5 saffron, and 7 white pepper samples) was evaluated, using HPLC and IAC. Aflatoxin B1 was detected in 43% of the samples, whereas G2, G1, and B2 were observed in none of the samples (7).

Aflatoxin level was evaluated in 91 samples (i.e., 70 powdered red pepper, 6 black pepper, 5 white pepper, 5 spice mix, and 5 chili samples) in Hungary, using HPLC and IAC purification (10). Overall, 18 out of 70 ground red pepper samples contained aflatoxin, seven of which had a concentration exceeding the maximum recommended level (5 μg/kg) (10).

Moreover, aflatoxin in cinnamon was studied, ground red pepper, and chamomile samples in Turkey. Among 100 samples, aflatoxin concentration was <0.025, 0.025–5, and > 5 μg/kg in 32, 50, and 18 samples, respectively (6). Additionally, in Italy was revealed aflatoxin contamination in pepper (range: 0.57–30.7 μg/kg) (11).

The level of aflatoxin was determined in four types of chili pepper and showed a value above the standard limit in three species (12). Moreover, AFB1 was detected in 58 (62%) out of 93 organic spice samples and 32 (86%) out of 37 organic herbal samples. AFB1 level in 41 (44%) out of 93 organic spice samples and 21 (57%) out of 37 organic herbal samples was above the standard limit European Union (3).

Aflatoxin exposure is high in Iran. Since livestock feeding by contaminated bread is one of the potential ways for milk contamination, control and reduction of the aflatoxin contamination by appropriate production process, storage condition and livestock feeding is essential. Pistachio, one of the most important exporting products of this country, was threatened by this contamination and to continue the export of it, regulations against aflatoxin contamination are important (13).

In different regions and states of Iran several generations and species of toxigenic fungi and aflatoxins had been detected in various types of food, air, and equipment. Farm-management and food-storage functions are essential at decreasing food-processing times, and these efforts can prevent or minimize the toxin in agriculture, industry, and food-product manufacturing to promote human and animal health (14–16).

Inter- and intra-species differences were observed in susceptibility of the major Iranian cereals as well as spices tested to *A. parasiticus* growth and aflatoxin production. Therefore, recommended studies to determine the resistance markers of selected cultivars of Iranian cereals (17).

## Conclusion

Aflatoxin contamination in spices, even at low levels, can be a serious threat, given the prevalent use of spices (either raw or not) in food preparation. Therefore, regular monitoring of spices, especially red pepper, is highly recommended, considering the popular use of spices around the world.

## Ethical considerations

Ethical issues (Including plagiarism, informed consent, misconduct, data fabrication and/or falsification, double publication and/or submission, redundancy, etc.) have been completely observed by the authors.
